# Molecular profiling of cutaneous squamous cell carcinomas and actinic keratoses from organ transplant recipients

**DOI:** 10.1186/1471-2407-13-58

**Published:** 2013-02-05

**Authors:** Liesbeth Hameetman, Suzan Commandeur, Jan Nico Bouwes Bavinck, Hermina C Wisgerhof, Frank R de Gruijl, Rein Willemze, Leon Mullenders, Cornelis P Tensen, Harry Vrieling

**Affiliations:** 1 Department of Toxicogenetics, Leiden University Medical Center, PO Box 9600, 2300, RC Leiden, the Netherlands; 2 Department of Dermatology, Leiden University Medical Center, PO Box 9600, 2300, RC Leiden, the Netherlands

**Keywords:** Cutaneous squamous cell carcinoma, Non-melanoma skin cancer, Actinic keratosis, Organ transplant recipient, Gene expression, Genomic profiling

## Abstract

**Background:**

The risk of developing cutaneous squamous cell carcinoma (SCC) is markedly increased in organ transplant recipients (OTRs) compared to the normal population. Next to sun exposure, the immunosuppressive regimen is an important risk factor for the development of SCC in OTRs. Various gene mutations (e.g. TP53) and genetic alterations (e.g. loss of CDKN2A, amplification of RAS) have been found in SCCs. The aim of this genome-wide study was to identify pathways and genomic alterations that are consistently involved in the formation of SCCs and their precursor lesions, actinic keratoses (AKs).

**Methods:**

To perform the analysis in an isogenic background, RNA and DNA were isolated from SCC, AK and normal (unexposed) epidermis (NS) from each of 13 OTRs. Samples were subjected to genome-wide expression analysis and genome SNP analysis using Illumina’s HumanWG-6 BeadChips and Infinium II HumanHap550 Genotyping BeadChips, respectively. mRNA expression results were verified by quantitative PCR.

**Results:**

Hierarchical cluster analysis of mRNA expression profiles showed SCC, AK and NS samples to separate into three distinct groups. Several thousand genes were differentially expressed between epidermis, AK and SCC; most upregulated in SCCs were hyperproliferation related genes and stress markers, such as keratin 6 (KRT6), KRT16 and KRT17. Matching to oncogenic pathways revealed activation of downstream targets of RAS and cMYC in SCCs and of NFκB and TNF already in AKs. In contrast to what has been reported previously, genome-wide SNP analysis showed very few copy number variations in AKs and SCCs, and these variations had no apparent relationship with observed changes in mRNA expression profiles.

**Conclusion:**

Vast differences in gene expression profiles exist between SCC, AK and NS from immunosuppressed OTRs. Moreover, several pathways activated in SCCs were already activated in AKs, confirming the assumption that AKs are the precursor lesions of SCCs. Since the drastic changes in gene expression appeared unlinked to specific genomic gains or losses, the causal events driving SCC development require further investigation. Other molecular mechanisms, such as DNA methylation or miRNA alterations, may affect gene expression in SCCs of OTRs. Further study is required to identify the mechanisms of early activation of NFκB and TNF, and to establish whether these pathways offer a feasible target for preventive intervention among OTRs.

## Background

Non-melanoma skin carcinomas, comprising cutaneous squamous cell carcinoma (SCC) and basal cell carcinoma (BCC), are the most common malignancies in fair-skinned Caucasians. The risk of developing cutaneous SCC is markedly increased (100-fold) in organ transplant recipients (OTRs) compared to the normal population [1,2] and is associated with the immunosuppressive therapy needed to prevent graft rejection [3]. Moreover, some studies have suggested that SCCs from OTRs display a more aggressive behavior [4].

Like other cancers, the development of cutaneous SCC is thought to be a multi-step process, involving sequential acquisition of genetic changes. Sun (UVR) exposure is the principal carcinogen inducing both immunologic tumor-tolerance and DNA damage that can lead to mutations in oncogenes and tumor suppressor genes (TSGs) [5,6]. Other risk factors include impaired immune surveillance, as seen in OTRs, and human papillomavirus (HPV) infections [7]. However, the oncogenic potential of HPV in cutaneous SCCs remains unclear [8]. In a recent transcriptome sequencing study no evidence of active viral gene expression was found in large group of SCCs [9].

Cutaneous SCCs are thought to develop from precursor lesions, actinic keratoses (AKs). Even though most AKs (80-99 %) do not progress to SCC, there is still both histological and molecular evidence to support this hypothesis (reviewed by C.J. Ko [10]). UVR-related mutations inactivating the TP53 tumor suppressor gene are very common in SCCs and AKs, both in immunocompetent patients and OTRs [5,11]. TP53 mutations are thought to occur very early in the development of skin cancer, since they are already present in microscopic clusters of keratinocytes in sun-exposed human skin [12]. These clusters are thought to precede tumor formation [13] and are more frequent in OTR compared to immunocompetent patients [14]. However, not every SCC contains a mutated TP53 gene [5].

Other common genetic changes found in cutaneous SCC development are the inactivation of the CDKN2A tumor suppressor gene (via mutation, promoter methylation and/or chromosomal loss) and mutation and/or amplification of RAS genes (reviewed in Boukamp et al. [15]). Both oncogenic changes are associated with the progression of AK to SCC. Also, (over-) expression of RAS downstream proteins (MAPKs and cyclins) has been reported in a subset of SCCs [16].

At the chromosomal level, several studies have shown that cutaneous SCCs can display complex karyotypes with large numbers of allelic imbalances [17-19]. Moreover, widespread gains and losses of chromosomal fragments have been reported to be already present in AKs [17,20].

The aim of our study was to identify genes and/or pathways that are consistently involved in the development of cutaneous SCC and their precursor lesions, AKs in OTRs. Thus far, the genome-wide transcriptional profiling studies on SCCs are diverse in nature, show little overlap in differentially expressed genes (DEGs), have limited sample size, use cell lines and do not integrate genomic and expression data [21-24]. This genome-wide study assesses in parallel both RNA and DNA alterations in a sizeable well-defined group of OTRs presenting with both AKs and SCCs. A better understanding of the pathological mechanisms leading to skin carcinomas in these patients could lead to novel molecular targets for effective intervention.

## Results

### Patient material

Patients were selected from the large group of OTRs that visit the dermatology clinic of the LUMC at a regular basis to provide a well-documented sample series for our study. Moreover from these OTRs it was possible to select both SCCs and AKs as well as normal skin (NS) to circumvent inter-patient variability. Fifteen OTRs (7 females and 8 males) participated in this study (Table 1). The average age of the patients when the selected SCC was diagnosed was 59 years (range 47–74 years). Side-matched normal skin in OTRs often already contains a fair amount of UV damage, a phenomenon described as ‘field cancerisation’ [25,26]. Therefore, (UV-unexposed) skin from the lower back was chosen as normal skin and served as internal negative control. Of patient 42, only SCC and AK samples were available, and for patient 15 only a SCC sample could be obtained. Samples of these two patients were only included in the Genome-wide expression analysis (GWEA). All patients received immunosuppressive drugs (Table 1).

**Table 1:** Patient and tumor characteristics

**Patient characteristics**					**SCC**	**AK**^**d**^	**NS**^**d**^	**Peripheral blood**
**Patient**	**Gender**^**a**^	**Age**^**b**^	**Transplanted organ**	**Drugs**^**c**^	**location**	**location**	**location**	**Yes/No**
P-3	M	56	Kidney	MMF, P	Upper leg	Forearm	Lower back	Yes
P-5	M	72	Kidney	Aza, P	Lower arm	Forearm	Lower back	Yes
P-11	F	56	Kidney	Aza, P	Tibia	Forearm	Lower back	Yes
P-15	M	62	Kidney	P	Lower arm	n.a.	n.a.	No
P-18	M	51	Kidney-pancreas	Aza, P	Dorsal hand	Forearm	Lower back	Yes
P-24	M	74	Kidney	Aza, P	Lower leg	Forearm	Lower back	Yes
P-27	F	56	Liver	Tacroli-mus, P	Shoulder	Forearm	Lower back	Yes
P-33	F	66	Kidney	Aza, P	Shoulder	Forearm	Lower back	Yes
P-38	F	47	Kidney	Aza, P	Shoulder	Forearm	Lower back	Yes
P-39	F	65	Kidney	Aza, P	Lower leg	Upper arm	Lower back	Yes
P-40	M	60	Kidney	MMF, P	Dorsal hand	Forearm	Lower back	Yes
P-41	M	61	Kidney	Aza, P	Dorsal hand	Forearm	Lower back	Yes
P-42	F	48	Kidney	Aza, P	Dorsal hand	Dorsal hand	n.a.	No
P-44	M	62	Kidney	Aza, P	Dorsal hand	Forearm	Lower back	Yes
P-57	F	53	Kidney	Aza, P	Tibia	Forearm	Lower back	Yes

^a^ F: female; M: male.

^b^ Age when SCC used for study was diagnosed.

^c^ Immunosuppresive drugs at time of SCC diagnosis. MMF: mycophenolate mofetil; P: prednisone; Aza: azathioprine.

^d^ n.a = not available for study.

### Genome-wide SNP analysis of SCCs and AKs

**Figure 1:**
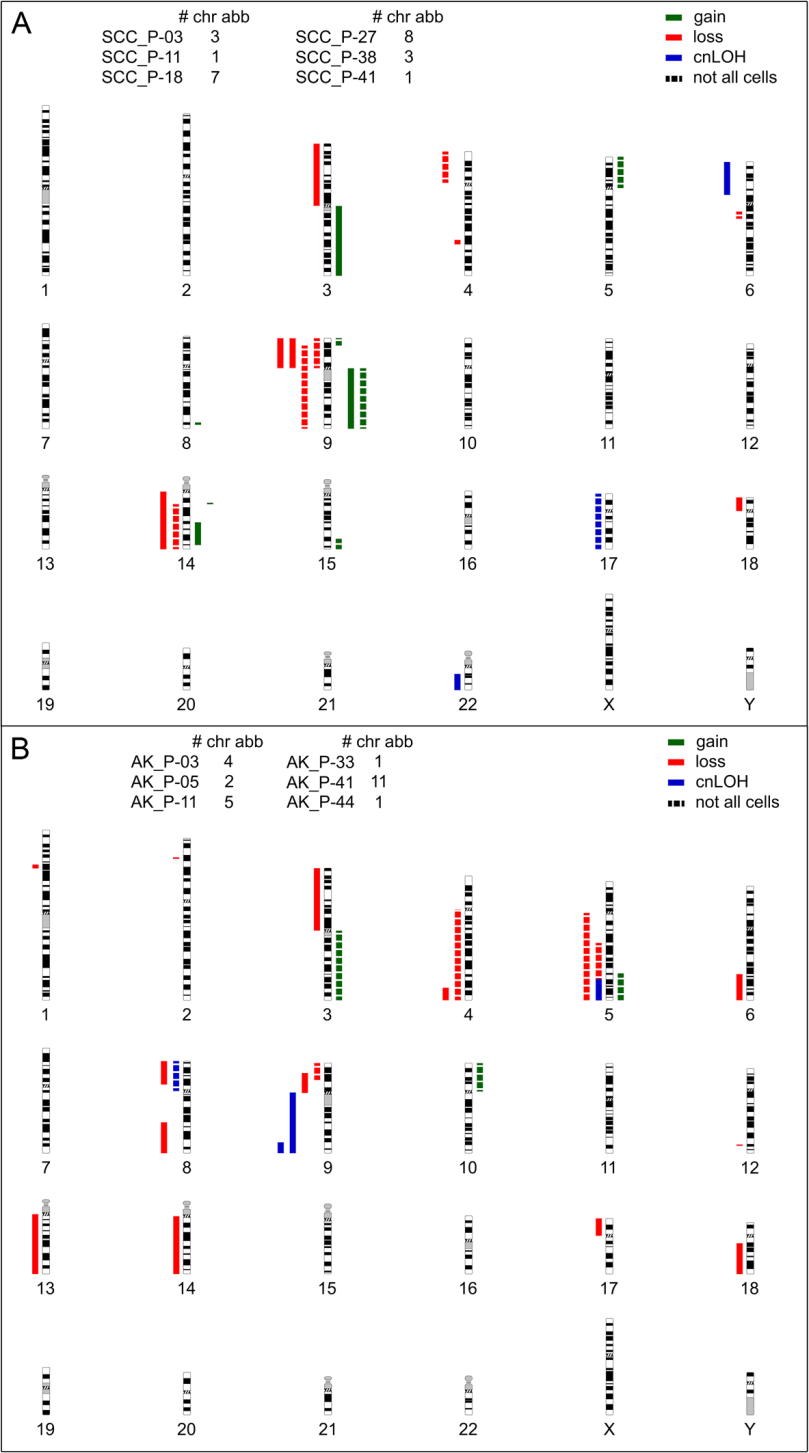
**Graphical overview of chromosomal aberrations in SCCs and AKs.** Idiograms summarizing chromosomal aberrations in SCCs **(A)** and AKs **(B)** compared to the patient matched normal control. LOH events are shown to the left of the chromosomes in either red (physical loss) or blue (copy-neutral LOH (cnLOH)). Gains are indicated on the right of the chromosomes in green. Dotted lines represent aberrations that were not present in all tumor cells of a sample.

Thirteen SCC samples and eleven AKs were subjected to genome-wide SNP array analysis together with the corresponding normal DNA from peripheral blood. Results are summarized in Table 2 and Figure 1. Six of thirteen SCCs showed chromosomal aberrations, ranging from a single loss of 9p to multiple losses and gains (amplification/duplication) (Table 2, Figure 1A). Copy number neutral loss of heterozygosity (LOH) was observed in 3 SCCs. Overall, more LOH than gain was seen (14 vs. 9). Also large deletions of (almost) whole chromosome arms were more frequent than small deletions (<40 Mb; 12 vs. 8). In several tumors the detected aberrations were not consistently present in >70% of the tumor sample (i.e. not a full signal of loss or gain), suggesting tumor heterogeneity (Table 2). Four out of thirteen (30%) SCCs demonstrated loss of 9p21 where CDKN2A is located.

**Table 2:** Overview of chromosomal aberrations in SCCs and AKs

	**Type of aberration**	**Cytoband**	**Mb location**	**Size (Mb)**	
SCC_P-03	Loss	4q28.3	1:136,448,440 - 136,988,409	0.54	*
	Gain	5p			*
	Gain	14q23.1-32.2	14:57,558,740 - 96,468,442	38.9	*
SCC_P-11	Gain	9q			*
SCC_P-18	Gain	8q24.22-24.23	8:132,122,430 - 139,467,653	7.3	*
	Gain	14q13.3	14:71,054,120 - 73,596,540	2.5	
	Gain	9p23-tel	9:1–12,039,121	12	*
	Loss	9p23-cen		~40	*
	Loss	9q			*
	Loss	14q13.3-tel		~70	*
	cnLOH	17			*
SCC_P-27	Loss	3p			
	Gain	3q			
	Loss	4p12-tel	4:1–46,377,946	46,4	*
	Gain	5q25.3-tel	5:83,159,010 - 100,338,915	17.1	*
	Loss	6q13-14.1	6:75,600,000 - 76,627,715	1.0	*
	Loss	9p			
	Gain	9q			
	cnLOH	18p			
SCC_P-38	cnLOH	6p21.31-tel	6:1–35,593,684	35.5	
	Loss	9p			
	Loss	14q			
SCC_P-41	cnLOH	22q12.1-tel	22:24,348,300 - 49,554,696	25.2	
AK_P-03	Loss	4q			*
	Loss	5q15-31.1	5:96,745,240 - 133,072,806	36.3	*
	cnLOH	5q31.1-tel	5:133,268,490 - 180,857,816	47.2	*
	cnLOH	9q			*
AK_P-05	Loss	5q			*
	Loss	9p21.1-tel	9:1–33,109,143		*
AK_P-11	Loss	2p22.2-25.1	2:11,690,000 - 36,950,000	25.3	*
	cnLOH	8p			*
	Loss	13q			*
	Loss	17p			*
	Loss	18q12.1-tel	18:26,013,440 - 76,117,152	50.1	*
AK_P-33	Loss	6q22.31-tel	6:122,757,319 - 170,975,699	48.2	*
AK_P-41	Loss	1p32.3	1:54,783,720 - 55,100,840	0.4	*
	Loss	3p			
	Gain	3q			*
	Loss	4q33-tel	4:171,450,620 - 191,411,215	20.0	
	Gain	5q31.2-tel	5:138,387,890 - 180,857,851	42.5	*
	Loss	8p12-tel	8:1–38,377,990	38.4	
	Loss	8q22.1	8:95,250,740 - 96,376,326	1.3	
	Loss	9p22.2-cen	9:17,230,530 - 50,549,240	33.3	
	Gain	10p	10:1–40,144,085	40.1	*
	Loss	12q24.23		0.5	
	Loss	14q			
AK_P-44	cnLOH	8q33.3-tel	8:126,444,040 - 137,979,818	11.4	

* heterogeneous chromosomal aberration: observed in less than 70% of the tumor sample.

Analysis of AK samples showed chromosomal aberrations in 6 of 11 samples, ranging from a single up to multiple (11) aberrations in an affected sample (Table 2, Figure 1B). Again, more losses and copy number neutral LOH than gains were seen (21 vs. 3). Generally, the aberrations observed in AKs, were less extensive than those in SCCs. No correlation was detected among the AKs regarding the extent of chromosomal damage and the relative level of dysplasia. However, similar to SCCs, several AKs demonstrated genetic heterogeneity.

Notably, there was no clear correlation between the number and type of aberrations found in the AK and SCC from the same OTR. However the number of samples in each group was not large enough to perform any statistical analysis.

### Cluster and single-gene analyses of expression profiles of SCCs and AKs

**Figure 2:**
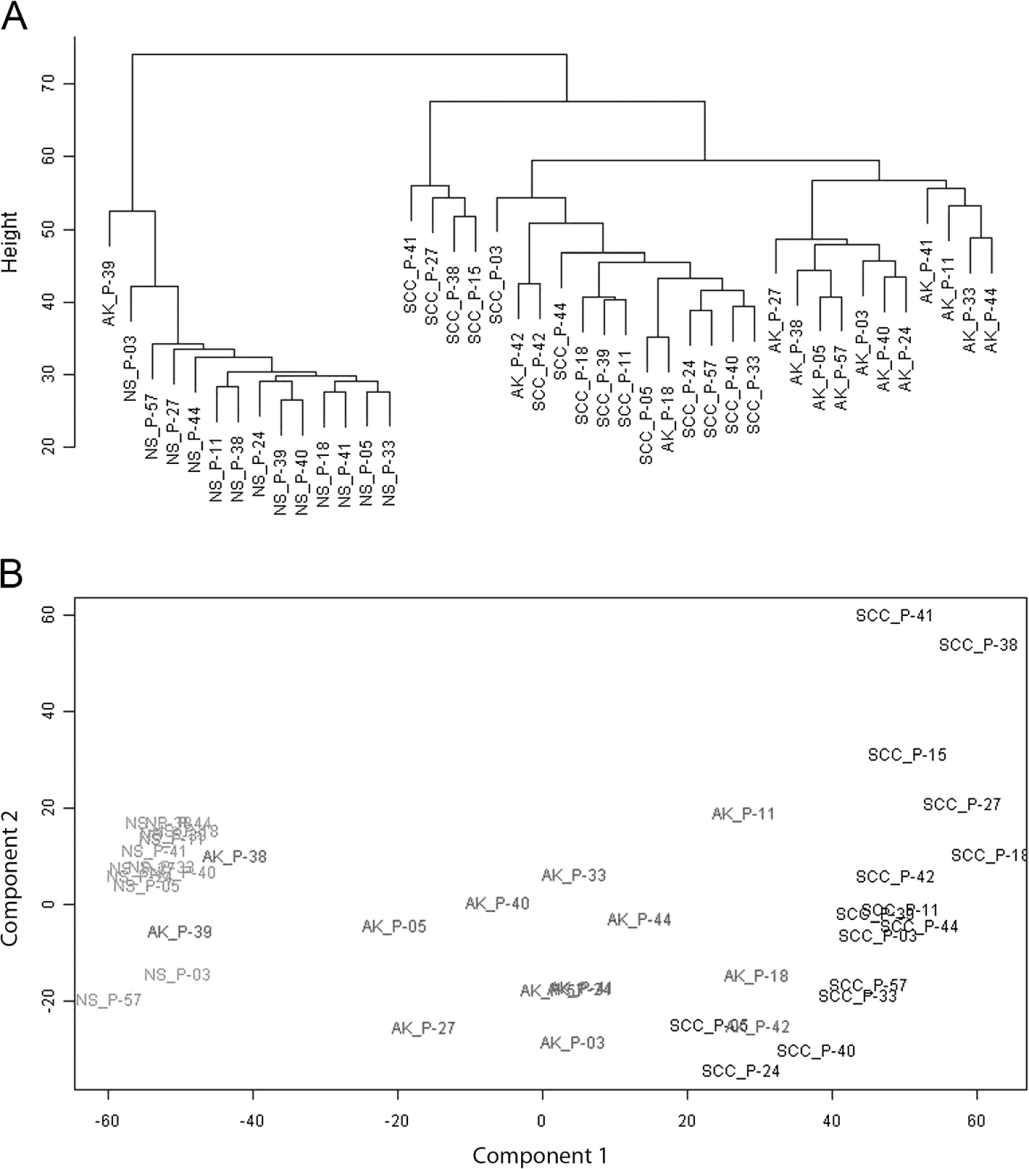
**Cluster analysis. (A)** Dendogram of unsupervised hierarchical cluster analysis of all samples. Clustering was based on 10,100 probes for which the average expression among all samples showed a standard deviation ≥ 0.15. Samples are labeled by sample group (NS/AK/SCC) and patient number (P-#). **(B)** Scatter plot of the first two components from PCA based on 6,104 probes for which the average expression among all samples showed a standard deviation/mean ≥ 0.1. Labels indicate the position for each sample (NS/AK/SCC) of a certain patient (P-#).

Fifteen SCC, fourteen AK and thirteen NS samples were included in the genome wide expression analysis (GWEA). Filtering for annotated and expressed genes resulted in 15,969 probes for further analyses. Subsequent hierarchical clustering, based on expression levels, resulted in two very distinct groups (Figure 2A), separating normal and tumor samples. The tumor samples were divided in three sub-clusters in one arm of the dendogram. Principal component analysis also demonstrated good separation of normal and tumor samples, already in the first component (Figure 2B). In the second component, AKs could be separated from SCCs. No correlation was seen with the type of organ transplanted. However, since 13 of 15 patients were kidney recipients, no definite conclusions can be made about this matter. The same could be concluded for the immunosuppressive regimen (data not shown).

Statistical analysis of the expression data was performed using limma. The following comparisons between sample groups were made: SCC vs. NS, SCC vs. AK and AK vs. NS. For each comparison a FDR <1% was used, and the comparisons were made with several log_2_ transformed fold change (log_2_FC) restrictions, namely log_2_FC > 0.5, log_2_FC > 1.0 and no log_2_FC-restriction (Table 3). With log_2_FC > 0.5, 2,087 probes, representing 2,009 genes, were differentially expressed between SCC and NS (Additional file 1). Among the upregulated genes in SCC were several genes known to be involved in keratinocyte differentiation and skin cancer, including keratins (e.g. KRT16, KRT17 and KRT6), matrix metalloproteinases (MMPs), S100 molecules and small proline-rich proteins (SPRRs). Most of these genes were highly overexpressed with a log_2_FC > 2.0. The cutaneous T-cell attracting chemokine, CCL27, was the most downregulated gene in SCCs compared to normal skin (log_2_FC = − 4.2).

**Table 3:** Overview of differentially expressed probes (DEPs) between the different sample groups

	**SCC vs. NS**	**AK vs. NS**	**SCC vs. AK**	**‘Progression’**
# of samples	15 vs. 13	14 vs. 13	15 vs. 14	
# of DEPs with FDR < 0.01	6820^*^ (3333/3487)	3734 (1554/2180)	2131 (1088/1043)	658 (262/396)
# of DEPs with FDR < 0.01 and log_2_FC > = 0.5	2087 (1087/1009)	977 (526/451)	571 (289/282)	180 (90/90)
# of DEP with FDR < 0.01 and log_2_FC > = 1.0	639 (370/269)	219 (167/52)	145 (81/64)	40 (32/8)

^*^ Total number of DEPs (number of upregulated DEPs/number of downregulated DEPs).

With log_2_FC > 0.5, 977 probes (947 genes) were differentially expressed between AK and NS samples and expression of 571 probes (547 genes) was significantly different between SCCs and AKs (Additional file 1).

**Figure 3:**
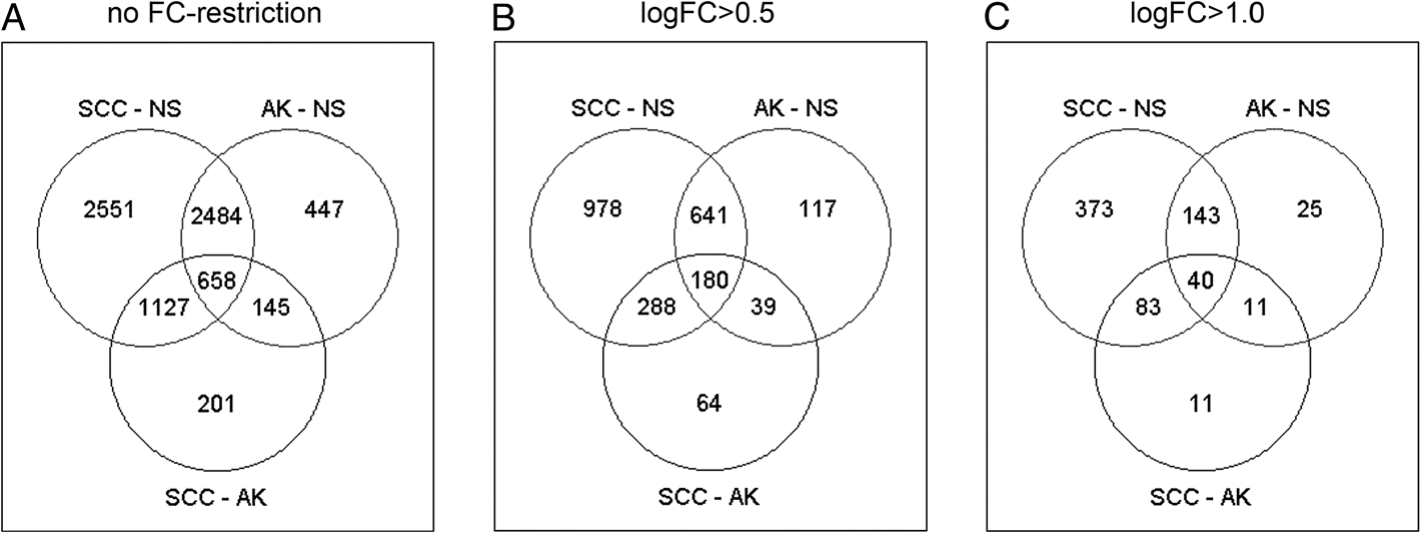
**Venn diagrams of differentially expressed probes (DEPs) from different comparisons within the three sample groups; NS, AK and SCC.** The FDR was set at <1%. **(A)** Differentially expressed probes with FDR < 1%. **(B)** DEPs with log_2_FC > 0.5 and FDR < 1%. **(C)** DEPs with log_2_FC > 1.0 and FDR < 1%.

In total for the log_2_FC > 0.5 analysis, 180 probes were present in all three comparisons as visualized by a VennDiagram (Figure 3, Additional file 1). These probes represented 173 genes that were consistently up- or downregulated during the development of SCC from NS, via AK, and can be considered as genes involved in gradual skin cancer progression.

Since SCC, AK and NS samples were available from each patient, we investigated whether the differentially expressed genes between sample groups differed per patient. To this end, a patient factor was included in the design of limma; however this did not change the results (data not shown).

### Geneset enrichment analyses of expression of SCCs and AKs

In addition to single-gene analysis, affected molecular pathways were investigated employing gene set enrichment analysis (GSEA) using two different approaches. Firstly, differentially expressed probes between the sample groups from the limma analysis with log_2_FC > 0.5 (Additional file 1) were analyzed for gene set enrichment with DAVID Bioinformatic Resources. All annotated and expressed probes (n = 15,969) were used as background list. Results are shown in Additional file 2. Genes involved in epidermal development and keratinocytes were among the most enriched GO terms of the biological processes (BP) node in all comparisons. Also KEGG pathways were analyzed by DAVID. When comparing SCC with AK, genes involved in “focal adhesion” and “pathways in cancer” were enriched among the DEGs (Additional file 2).

**Figure 4:**
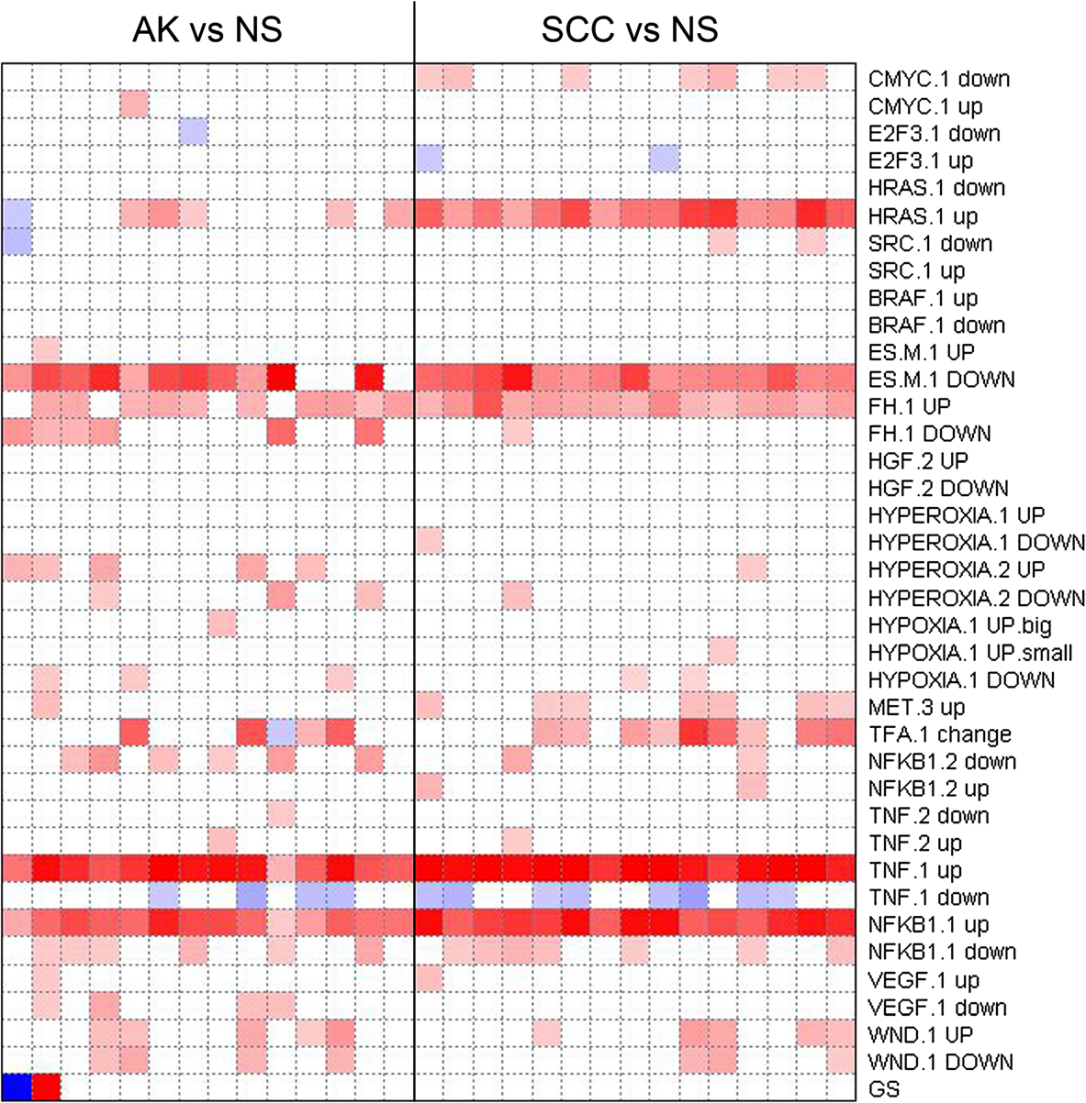
**Results Parametric geneset enrichment analysis (PGSEA).** Gene expression profiles derived from AK (n = 14) and SCC (n = 15) samples were compared with gene expression profiles derived from normal skin (NS, n = 13) samples and analyzed using PGSEA for the gene lists that contain genes responsive to oncogenes or for indicated pathways. The genes that show increased expression to NS for each pathway are indicated with ‘up’. List of genes that show decreased expression relative to control cells for each pathway are indicated with ‘down’ [28]. The resulting t-statistic for each gene list was plotted (−10 < t < 10), p < 0.005); red squares represent significant number of genes in the list with increased expression in tumor samples (AK or SCC) relative to NS; blue squares represent a significant number of genes in each list with decreased expression.

In the second GSEA approach, the entire dataset was analyzed with the PGSEA package (Figure 4). In this analysis expression profiles of AKs and SCCs were compared to those of NS. PGSEA analysis demonstrated that genes upregulated by RAS were enriched in SCC compared to NS, whereas this was not seen in AK. For both SCC and AK genes positively regulated by TNF, NFκB and fumarate-hydratase (FH, known to be involved in aggressive renal papillary cancers) [27] were enriched. Genes regulated by the oncogenes BRAF and SRC were not enriched in tumors compared to NS.

In addition to GSEA, the results from the limma analysis (log_2_FC > 0.5, FDR < 0.01, Additional file 1) were subjected to oPOSSUM analysis to investigate if the DEGs were controlled by specific transcription factor (TFs). The TFs whose transcription factor binding sites (TFBSs) were found enriched are listed in Table 4. In the list TFs of upregulated genes, the REL transcription factor class (incl. RELA and NFκB1) was overrepresented. These results correspond with the PGSEA, where genes controlled by NFκB1 were upregulated in SCC and AK (Figure 3). The forkhead (incl. FOXF2 and FOXD1) and ZH-finger (incl. SP1 and MZF1) TF classes were overrepresented in the TFBS of downregulated genes.

**Table 4:** Results oPOSSUM analysis of overrepresented transcription factors in the differentially expressed genes from limma (logFC > 0.5, FDR < 0.01)

**TF**	**TF class**	**Target genes**	**Z-score**^**a**^	**Fisher score**^**b**^
**SCC vs NS**				
*Up- and downregulated genes (1514 of 2036 genes included*^*c*^*)*				
SRF	MADS	107	12.25	7.38E-05
Hand1-Tcfe2a	bHLH	1148	10.30	3.31E-06
RELA	REL	576	8.01	1.21E-06
SP1	ZN-FINGER, C2H2	1036	7.80	1.34E-09
SPIB	ETS	1374	5.97	4.72E-06
*Upregulated genes (736 of 1039 genes included)*				
SRF	MADS	62	16.93	5.02E-05
RELA	REL	291	11.68	2.66E-04
Hand1-Tcfe2a	bHLH	565	9.30	1.53E-02
ELF5	ETS	658	9.25	2.38E-05
REL	REL	450	8.40	2.86E-05
*Downregulated genes (758 of 1004 genes included)*				
FOXF2	FORKHEAD	221	12.93	1.52E-06
SP1	ZN-FINGER, C2H2	536	11.03	1.12E-08
FOXD1	FORKHEAD	459	8.409	2.16E-06
Foxq1	FORKHEAD	349	7.176	1.47E-06
TP53	P53	3	6.687	1.04E-01
**AK vs NS**				
*Up- and downregulated genes (695 of 960 genes included)*				
SP1	ZN-FINGER, C2H2	494	16.58	1.32E-08
MZF1_1-4	ZN-FINGER, C2H2	644	14.28	2.43E-07
SRF	MADS	56	13.50	1.32E-04
Cebpa	bZIP	437	10.90	1.99E-05
TEAD1	TEA	217	10.80	8.74E-05
MZF1_5-13	ZN-FINGER, C2H2	527	10.37	1.59E-08
*Upregulated genes (364 of 515 genes included)*				
SRF	MADS	35	19.82	8.10E-05
ELF5	ETS	319	11.05	2.04E-04
RELA	REL	150	10.03	1.60E-04
SPIB	ETS	338	9.25	2.28E-04
TLX1-NFIC	HOMEO/CAAT	32	9.00	5.07E-03
*Downregulated genes (695 of 960 genes included)*				
SP1	ZN-FINGER, C2H2	248	17.04	1.63E-07
MZF1_1-4	ZN-FINGER, C2H2	309	11.7	3.74E-04
Arnt-Ahr	bHLH	289	11.49	2.25E-09
Cebpa	bZIP	228	11.18	5.86E-07
Roaz	ZN-FINGER, C2H2	130	10.88	2.11E-05
**SCC vs AK**				
*Up- and downregulated genes (412 of 553 genes included)*				
RREB1	ZN-FINGER, C2H2	38	11.29	1.24E-03
FOXF2	FORKHEAD	111	10.99	7.16E-03
SRF	MADS	36	10.01	4.10E-04
RELA	REL	163	9.239	8.26E-04
TEAD1	TEA	120	8.031	2.46E-02
*Upregulated genes (209 of 283 genes included)*				
SRF	MADS	24	15.39	6.72E-05
RELA	REL	85	11.99	5.32E-03
Fos	bZIP	157	10.98	3.68E-04
NF-kappaB	REL	108	8.39	2.22E-04
REL	REL	129	7.91	1.93E-03
*Downregulated genes (204 of 271 genes included)*				
FOXF2	FORKHEAD	70	22.37	2.55E-05
FOXI1	FORKHEAD	126	10.74	7.22E-02
RREB1	ZN-FINGER, C2H2	22	9.86	1.81E-03
T	T-BOX	28	8.77	5.00E-03
Foxd3	FORKHEAD	129	7.91	5.37E-02
**Progression genes**				
*Up- and downregulated genes (128 of 172 genes included)*				
RELA	REL	54	10.18	1.00E-02
SP1	ZN-FINGER, C2H2	94	9.66	1.76E-03
FOXF2	FORKHEAD	36	7.30	5.18E-02
NFKB1	REL	35	6.21	1.09E-02
MZF1_1-4	ZN-FINGER, C2H2	118	6.11	3.15E-02
*Upregulated genes (53 of 85 genes included)*				
RELA	REL	23	14.0	5.41E-02
NFKB1	REL	19	9.78	2.48E-03
SRF	MADS	6	8.09	3.82E-02
TLX1-NFIC	HOMEO/CAAT	7	7.65	2.27E-02
MIZF	ZN-FINGER, C2H2	11	7.11	3.21E-02
*Downregulated genes (75 of 87 genes included)*				
FOXF2	FORKHEAD	29	14.63	6.46E-04
FOXI1	FORKHEAD	48	11.42	1.14E-01
T	T-BOX	13	8.14	7.45E-03
Foxa2	FORKHEAD	50	8.02	4.40E-02
SP1	ZN-FINGER, C2H2	56	7.60	7.94E-03

^a^ The Z-score determines whether a TFBS occurs more frequently in the set of co-expressed genes compared to pre-computed background set provided by the package.

^b^ The Fisher score (one-tailed Fisher exact) is calculated to determine the probability of non-random association between the co-expressed genes and the TFBS site of interest.

^c^ The number of genes in the differentially expressed gene set that could be aligned to the pre-computed background.

For more details on the oPOSSUM analysis: S.J. Ho Sui et al., (2005) Nucleic Acid Res., 33: 3154–64.

### Comparison of genome-wide SNP analysis and GWEA

Expression levels of both AKs and SCCs were compared to those of NS based on their chromosomal location to identify any regional chromosomal expression bias using the *reb* package [28]. Since at the genomic level only a few, non-recurrent chromosomal alterations were found, we compared the results of the genome-wide SNP analyses with those of the regional expression level results per tumor. However, in only very few regions where at the SNP level alterations were seen the expression level of genes in this region were affected too (data not shown).

### Validation of the GWEA with QPCR

**Figure 5:**
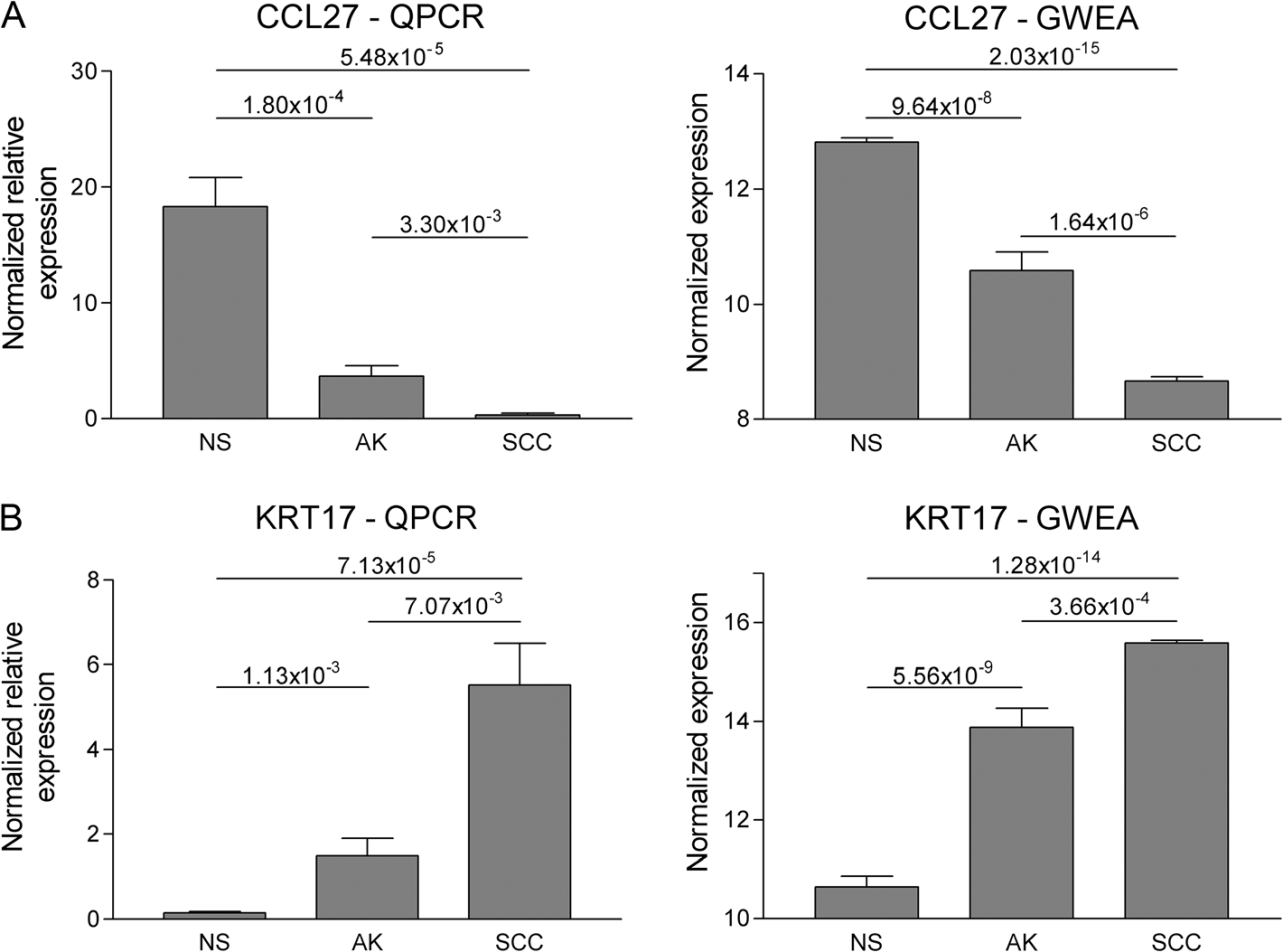
**QPCR and genome-wide expression analysis (GWEA). (A)** Histograms showing the expression level of CCL27 in NS, AK and SCC samples measured by QPCR (left) and GWEA (right). **(B)** Histograms showing the expression level of KRT17 in NS, AK and SCC samples measured by QPCR (left) and GWEA (right). For QPCR the normalized relative expression level represents the expression of the gene of interest normalized to those of four reference genes. For the GWEA the normalized expression level represents the RSN normalized, VST transformed expression of the gene of interest.

To validate microarray results, 9 DEGs were selected based on their high log_2_FC, highly significant adjusted p-value, involvement in identified pathways from the GSEA and potential biological relevance for SCC development. Their expression was quantified by QPCR analysis in all patient samples. The expression of these 9 genes was normalized for the expression of 4 reference genes, which were selected based on their stable expression in the GWEA, using geNORM. Relative differences between sample groups were confirmed for all genes (Table 5, Figure 5), although fold changes were often higher in QPCR results compared to those from the GWEA (Table 5). This is probably due to the larger dynamic range of QPCR compared to array-based analysis.

**Table 5:** Results QPCR validation compared with the results from the genome-wide expression analysis

**QPCR validation experiment**												
	**Normalized relative expression**^**a**^						**Fold change and P-value Student’s*T*-test**^**b**^					
	**NS (n = 10)**		**AK (n = 13)**		**SSC (n = 15)**		**SCC vs NS**		**AK vs NS**		**SCC vs AK**	
**Gene**	**Median**	**SE**	**Median**	**SE**	**Median**	**SE**	**log**_**2**_**(FC)**	**p-value**	**log**_**2**_**(FC)**	**p-value**	**log**_**2**_**(FC)**	**p-value**
CCL27	18,45	2,53	2,46	0,91	0,11	0,14	−7,34	**5,48E-05**	−2,91	**1,80E-04**	−4,43	**3,30E-03**
KRT17	0,12	0,03	1,19	0,41	5,20	0,97	5,39	**7,13E-05**	3,26	**7,07E-03**	2,13	**1,13E-03**
MMP1	0,01	0,01	0,28	1,16	47,19	35,2	12,18	5,68E-02	4,80	**2,32E-01**	7,38	6,14E-02
MMP3	0,01	0,01	0,36	0,37	17,91	4,95	10,99	**3,03E-03**	5,36	4,07E-02	5,63	**4,31E-03**
MMP9	0,07	0,01	1,83	0,74	4,49	4,21	6,07	**1,82E-02**	4,77	**4,42E-03**	1,30	6,07E-02
MMP10	0,02	0,03	0,95	1,17	17,70	8,24	9,59	**2,69E-03**	5,38	4,53E-02	4,21	**5,16E-03**
PI3	0,02	0,01	2,01	1,67	10,62	6,23	8,95	**5,46E-03**	6,55	**7,71E-03**	2,40	**3,25E-02**
SERPINB4	0,004	0,002	1,99	6,2	18,79	15,7	12,33	**2,35E-02**	9,09	**3,96E-02**	3,24	1,46E-01
TUBB3	0,51	0,08	0,64	0,17	3,68	0,94	2,85	**1,17E-03**	0,32	**3,20E-01**	2,53	**1,78E-03**
**Genome-wide expression analysis**												
	**Normalized expression **^**c**^						**Results limma analysis**					
	**NS (n = 13)**		**AK (n = 14)**		**SSC (n = 15)**		**SCC vs NS**		**AK vs NS**		**SCC vs AK**	
**Gene**	**Median**	**SE**	**Median**	**SE**	**Median**	**SE**	**log**_**2**_**FC**	**Adj. p-val**	**log**_**2**_**FC**	**Adj. p-val**	**log**_**2**_**FC**	**Adj. p-val**
CCL27	12,92	0,02	10,74	0,10	8,60	0,03	−4,24	2,03E-15	−2,24	9,64E-08	−2,00	1,65E-06
KRT17	10,58	0,07	14,43	0,10	15,56	0,01	4,84	1,28E-14	3,16	5,56E-09	1,68	3,66E-04
MMP1	8,32	0,00	8,39	0,05	10,80	0,08	2,41	5,70E-10	0,22	5,65E-01	2,20	1,61E-07
MMP3	8,33	0,01	8,55	0,06	10,59	0,08	2,44	1,16E-09	0,50	1,59E-01	1,84	6,86E-06
MMP9	8,48	0,01	9,29	0,06	10,50	0,09	2,22	1,37E-07	1,05	8,50E-03	1,93	2,02E-06
MMP10	8,31	0,00	8,33	0,02	10,02	0,10	1,93	3,07E-07	0,09	8,41E-01	1,16	4,61E-03
PI3	9,16	0,05	13,60	0,14	15,27	0,04	5,68	1,33E-13	3,96	1,09E-08	1,72	3,44E-03
SERPINB4	8,34	0,02	10,22	0,13	12,04	0,10	3,81	5,15E-09	2,16	3,64E-04	1,65	6,04E-03
TUBB3	8,84	0,01	8,86	0,05	10,45	0,05	1,91	5,45E-11	0,23	3,66E-01	1,67	5,28E-08

^a^ Relative expression normalized to the expression level of four reference genes.

^b^ Bold values represent the comparisons that could be statistically confirmed in the QPCR validation experiment.

^c^ VST transformed RSN normalized data.

## Discussion

In the present study we performed genome-wide expression analysis on SCCs, AKs and NS from OTRs to identify common pathways involved in the cellular transformation from normal skin cells to AKs and eventually SCC. Data analysis disclosed large numbers of DEGs between NS, AKs and tumor samples and identified the activation of several known oncogenic pathways in SCC, some of which were also activated in AKs.

Our unique isogenic approach in this genome-wide profiling study on a well-documented sample series of OTRs, would circumvent the influence of any possible patient-specific differences on the analysis, including type of immunosuppressive drug used, and would allow identification of genes and pathways specifically involved in progression of skin carcinogenesis. During the last years, several genome-wide expression studies on SCC and AKs have been conducted [21-24]. Most studies focused on genes differentially expressed in SCCs compared to normal skin, although some also included AK samples and cell lines. Since the various studies used different gene expression platforms, tumor selection criteria and isolation and labeling methods, it was impracticable to combine data from these studies with our data into one large dataset and perform a meta-analysis. However, we do see considerable overlap in results obtained, especially after pathway en gene set analyses.

Cluster analysis showed that samples derived from the same patient did not cluster together, indicating that gene expression differences between sample groups (NS, AK and SCC) were larger than differences between patients. So, inter-patient variation did not discernibly influence the results of the expression analysis in our study. Furthermore, gene expression profiles of three AKs demonstrating (mild-to-) severe dysplasia (AK_P-11, AK_P-18 and AK_P42) showed more similarity to that of SCCs than to the other AKs (Figure 2). Even though the numbers were too small to make any statistically reliable statement, this result suggests that the progression of AK to SCC is a continuous process.

Limma analysis demonstrated that more than 40 % of the expressed and annotated probes were differentially expressed between SCC and NS; smaller numbers of DEGs were identified between AK and NS and between SCC and AK. When the analysis was restricted to probes with a log_2_FC > 0.5 or log_2_FC > 1.0 the number of differentially expressed probes reduced dramatically, indicating that it was possible to identify subtle but significant changes in expression level in many genes. However, the large number of DEGs hampered identification of genes crucial to SCC development. Moreover, at least for the comparison of SCC with normal skin, differential gene expression might also be caused by non-specific differences owing to increased proliferation and metabolic rate of fast-growing tumor cells compared to keratinocytes in the epidermis. Similar results were obtained previously, where many transcripts showed relatively small changes in expression level between SCC and sun-exposed skin [22]. Additional gene-targeted, approaches are needed to investigate which genes/pathways are crucial for AK formation and subsequent SCC development.

Next to single gene analysis, GSEA was performed to identify specific gene sets and pathways involved in SCC development. With the online resource tool DAVID both GO terms and KEGG pathways were analyzed. From the GO terms analysis, many gene sets involved in (epidermal) cell proliferation and differentiation were enriched in SCC and AK. These gene sets include genes encoding keratins, MMPs, S100 molecules and SPRRs, all (highly) upregulated in SCC and AK compared to normal skin. According to the KEGG analysis, several cancer pathways were enriched in SCC vs. AK or SCC vs. NS, and included ‘pathways in cancer’ and ‘small cell lung cancer’. These cancer pathways comprise many genes downstream of known oncogenes, like RAS and MYC.

Upregulation of downstream targets of RAS was also identified by PGSEA when comparing the profiles of SCC to NS, indicating that this pathway is activated in SCC. NFκB1 and TNF pathways were activated in both AKs and SCCs, suggesting that activation of these pathways is an early event in the development of AK and SCC. NFκB1 is part of the REL TF family, which also includes REL and RELA. Gene promoter analysis by oPOSSUM showed that upregulated genes in SCC and AK were enriched for TF binding sites for REL transcription factors. These TFs have shown to be important in both skin development and carcinogenesis [29,30]. The results from our oPOSSUM analysis concurred with those of a previous study [22].

Both PSGEA and DAVID demonstrated activated RAS signaling in SCC, but not in AK. Previous studies have suggested that RAS might be activated via amplification of the gene locus or by activating mutations (reviewed by Boukamp [15]). However, both copy number alterations (SNP analysis) and oncogenic hot-spot mutations in HRAS and KRAS (data not shown) were not found in our SCCs, suggesting other molecular events to be responsible for activating the RAS signaling. These might include overactivation of the epidermal growth factor receptor (EGFR) upstream of RAS, which is known to occur in a subset of cutaneous SCCs [31,32].

The accuracy of the GWEA was confirmed by validation experiments using QPCR. Differential expression could be confirmed for all nine genes tested. These genes were selected based on their log_2_FC and adjusted p-value, but also for their presumed biological relevance for the development to SCC. One of these genes, the keratinocyte-specific chemokine CCL27 was confirmed to be downregulated progressively in AK and SCC, corresponding to previous findings that CCL27 was downregulated in keratinocyte-derived tumors [33]. As CCL27 is involved in mediating cutaneous homing of T-lymphocytes [34], its downregulation could signify possible immune evasion by cutaneous SCCs. Furthermore, our GWEA results are in agreement with previous reports that CCL27 is downregulated through activation of the RAS-MAPK-signaling pathway in human skin tumors [33].

Several DEGs identified by GWEA concerned genes involved in epidermal differentiation. Upregulation of intermediate filament keratins KRT6, KRT16 and KRT17, concurred with our previous work in which we found these proteins abundantly expressed in cutaneous SCCs of OTRS, in contrast to normal skin [35]. Also a recent study by Hudson et al. with samples from immunocompetent patients, showed that genes involved in epidermal differentiation were amongst the most DEGs between SCC and normal skin [22].

Next to the genes important in epidermal differentiation, genes involved in extracellular matrix (ECM) organization were differentially expressed in SCCs compared to NS. Among others, QPCR confirmed MMP1, MMP3, MMP9 and MMP10 to be highly upregulated in SCC compared to normal skin, sometimes already in AK. Although MMPs are normally induced temporarily in response to exogenous signals, they are known to play a role in tumor progression and metastasis. MMP1, MMP3 and MMP10 are virtually absent in normal skin, but often found upregulated in cutaneous SCC, although not in AK [36,37]. Moreover, MMP9 has been shown to be involved in the early development of cutaneous SCC in immunosuppressed individuals [38]. Even though it is known that the different MMPs have separate functions [37], MMPs often cooperate in parallel and are regulated by specific and non-specific inhibitors, such as serine proteases such as chymase, plasmin and kallikrein, to achieve targeted ECM degradation. We were able to identify several kallikrein proteins upregulated in SCC compared to normal skin. Upregulation of plasmin and chymase was not found, however these might be inhibited by serpin peptidase inhibitors, such as SERPINB3 and SERPINB4. These genes, which are known to produce circulating SCC-antigens 1 and 2 respectively, were highly upregulated in SCCs in our study and have previously been reported to promote survival of SCC cells in vitro [39].

The SCCs and AKs from the OTRs investigated in this study displayed very few genomic changes. The careful selection of tumor material and the results of the GWEA and QPCR analyses, make us believe that this lack of observed changes is not caused by interference of tumor material with that of normal cells. Nor do we have any indication that many alterations remained undetected because of the Illumina Genotyping Beadchip platform we used. This platform has been successfully used in various other genomic profiling studies [40,41]. Our results from the genome-wide SNP analyses contrast reports on previous studies in which SCCs displayed highly complex karyotypes, including many gains and losses of entire chromosome arms [15,42]. Also in AKs chromosomal aberrations have been reported to occur [20]. However, more recently Purdie et al. demonstrated that SCCs could be separated in genetically distinct subpopulations related to the differentiation status of the tumor [19]. Our results coincide with the observation in this study that well-differentiated SCCs possess fewer genomic aberrations compared to moderate and poorly differentiated tumors, albeit that the absolute number of aberrations per SCC in our study was lower. Although no relationship was detected by Purdie et al. between the patient’s immune status and the number of aberrations observed, eight of nine tumor with 3 or less alterations originated from transplant patients. This observation and the results of our study suggest that perhaps the suppression of the immune system in OTRs may influence the development of SCCs in these patients. This phenomenon has been reported before by Rehman et al., who demonstrated that the LOH rate in SCCs from OTRs was less than half of that in SCCs from immunocompetent patients [43]. Moreover, there could also be a direct effect of the applied immunosuppressive drugs on the molecular pathogenesis of SCCs in OTRs, which has also been suggested before by others [44]. Larger compara-tive studies with SCCs from both OTRs and immunocompetent patients will be necessary to elucidate this matter further.

The results from our genome-wide SNP analysis are unexpected in view of the many changes in mRNA expression levels found in both SCCs and AKs compared to normal skin. Next to a role for immunosuppression as seen in OTRs, the many differences in gene expression observed might be attributable to epigenetic alterations, such as DNA methylation or changes in miRNA expression. The fact that many genes were differentially expressed between tumors and normal skin may be caused by demethylation of these genes or modulation of their transcription factors (e.g. (de)methylation or (de)phosphorylation or interference from miRNA).

## Conclusions

With this study we were able to demonstrate vast differences in gene expression profiles to exist between SCC, AK and NS from immunosuppressed OTRs. Moreover, we found that several pathways activated in SCCs were already activated in AKs, confirming the assumption that AKs are the precursor lesions of SCCs. These pathways include the NFκB1 and TNF pathways. RAS and MYC oncogenic pathways on the other hand appear to be specifically activated in SCC. Since the drastic changes in gene expression appeared unlinked to specific genomic gains or losses, the causal events driving SCC development require further investigation. The outcome of the current study also suggests that the early activation of NFκB1 and TNF pathways in the course of SCC development may offer opportunities for targeted preventive intervention in OTRs which may counter act “field cancerisation” by eradicating plaques formed by multiple AKs.

## Methods

### Patient material

Approval for the present studies was granted by the Leiden University Medical Center institutional review board. Patients were selected from the group of OTRs that are regularly seen at the dermatology clinic of the Leiden University Medical Center [45]. Patients with clinically suspected SCC were informed on the study and after informed consent was obtained, fresh frozen samples were obtained from the SCC. An AK on the forearm and unexposed normal skin (NS) from the buttock were taken and peripheral blood was drawn to serve as normal internal control in genome-wide SNP analysis. All clinical diagnoses were histologically confirmed. SCCs can be histologically categorized as “well”, “moderate” or “poorly differentiated” tumors, based on the degree of keratinization and cellular atypia [46]. All SCCs included in this study were classified as well-differentiated. Most AKs showed mild dysplasia, with the exception of AK_P-11 and AK_P-18, both demonstrating mild to severe dysplasia and AK_P-42 with severe dysplasia.

All samples were further handled in an anonymously coded fashion, following the medical ethical guidelines described in the Code ‘Proper Secondary Use of Human Tissue’ established by the Dutch Federation of Medical Sciences (http://www.federa.org).

### RNA and DNA isolation

RNA and DNA were isolated from SCC and AK biopsy samples that contained at least 70% tumor cells, as determined by haematoxylin and eosin stained frozen sections. To ensure that the RNA and DNA represent the same tumor cells/biopsy, the various cut sections were alternately used for RNA and DNA isolation, respectively. From the sample of unexposed NS the epidermis was removed for further processing by cryosectioning parallel to the outer surface of the skin biopsy (cut to a depth where opaque whiting of the remaining dermal surface was observed).

RNA was extracted from frozen material using the RNeasy Fibrous Tissue kit (Qiagen, Hilden, Germany), which included proteinase K treatment (10 min at 55°C) of the lysed sample in RLT-buffer and on-column DNase treatment. DNA from the tumor and skin samples was isolated using the Genomic-tips 20/G kit (Qiagen). A salting-out procedure was used to isolate genomic DNA from the peripheral blood samples [47].

Both RNA and DNA were quantified using a Nanodrop (NanoDrop technologies, Wilmington, CA) and RNA was further evaluated for degradation with the Lab-on-a-chip assay on the 2100 Bioanalyzer (Agilent Technologies, Santa Clara, CA, USA).

### Genome-wide SNP analysis

Genomic DNA from AKs, SCCs and their corresponding normal controls (peripheral blood) were subjected to genome-wide SNP analysis. 400 ng of DNA was assayed with the Infinium II Sentrix HumanHap550v3 duo Genotyping BeadChip (Illumina, San Diego, CA, USA), containing over 550,000 unique tag SNP markers. Hybridizations were performed at the Leiden Genome Technology Center according to the manufacturer’s instructions. Image analysis and quality control (call rate >98%) was performed in Illumina’s BeadStudio Version 3.2 software using the genotyping module. Each BeadChip was self-normalized using information contained within the array. Further analysis and visualization was performed with the Illumina Genome Viewer in Beadstudio. First, tumor data was paired with data from their corresponding normal control. Subsequently, examination of the log R ratio (signal intensity tumor/signal intensity normal control) and B allele frequency (BAF) was analyzed for the presence of copy number variations (CNVs) and/or loss of heterozygosity (LOH) in the tumor samples. By plotting the log R ratio across a chromosome, CNVs could be visualized by an increase or decrease in baseline signal. Secondly, the BAF was plotted across each chromosome to detect regions of LOH in tumor cells.

A region with LOH was defined as a stretch of heterozygous SNPs in normal DNA but homozygous in the matched tumor DNA.

### Genome-wide expression analysis (GWEA)

Gene expression profiles were obtained using HumanWG-6 v2 Expression BeadChips (Illumina). In brief, 100 ng of total RNA was converted to cDNA and subsequently labeled cRNA using the Ambion Illumina TotalPrep RNA Amplification kit (Ambion, Austin TX, USA) according to manufacturer’s instructions. The labeled cRNAs were hybridized overnight to the HumanWG-6 v2 BeadChips. After washing, the BeadChips were scanned using the Illumina BeadArray Reader to measure the fluorescence intensity of each probe.

Raw data was extracted from the BeadChip data files in Illumina’s BeadStudio Version 3.2 software using the gene expression module. Background subtracted data was further analyzed in R-based Bioconductor package, lumi (version 1.12.4) [48]. In lumi, the data was transformed (variance-stabilizing transformation (VST)) [49] and normalized (robust spline normalization (RSN)) [48], resulting in log-transformed normalized data. Several quality control plots of both the normalized and unnormalized data were generated in the lumi package. The R-package illuminaHumanv2.db (version 1.4.1) was used for annotation.

The data were purged of genes that did not meet the detection limit (expression-detection P-value >0.01) and/or were not annotated. This selection resulted in 15,969 probes, representing 13,848 genes that were subjected to further analysis.

Hierarchical clustering and principal component analysis (PCA) were used to cluster the samples based on their expression levels, using the bioDist package (version 1.18.0).

The limma package (version 3.2.3) [50] was used to identify differentially expressed genes (DEGs) between SCC, AK and NS. A Benjamini and Hochberg False Discovery Rate (FDR) of 1% was used as cut-off to select significant DEGs and correct for multiple testing.

Gene set enrichment analysis (GSEA) for enriched Gene Ontology (GO) terms and KEGG pathways was performed with the significantly DEGs from the limma analysis using DAVID Bioinformatic Resources v6.7 (http://david.abcc.ncifcrf.gov) [51]. Secondly, GSEA on the entire data set was performed using the parametric gene set enrichment analysis (PGSEA) and regional expression bias (reb) packages (version 1.14.0) [28].

To identify activation of transcription factors (TFs) in AKs and SCCs, the DEGs from the limma analysis were investigated for over-represented TF binding sites (TFBSs) using the online analysis tool oPOSSUM [52]. Only the vertebrate taxonomic supergroup was selected and for each comparison the top 5 TFs were selected.

Both the gene expression data and genome-wide SNP analysis have been deposited in the NCBIs Gene Expression Omnibus [53] as a SuperSeries with accession number GSE32979 (http://www.ncbi.nlm.nih.gov/geo/query/acc.cgi?acc=GSE32979).

### Quantitative RT-PCR (QPCR)

For 10 NS, 13 AK and 15 SCC samples sufficient amounts of total RNA were available for cDNA synthesis using the iScript™ cDNA Synthesis Kit (Bio-Rad, Veenendaal, the Netherlands) according to manufacturer’s instructions. Primers (Invitrogen, Breda, the Netherlands) were designed for nine genes of interest and four reference genes (Table 6). The reference genes were selected based on their stable expression in all samples in the GWEA. QPCR reactions were performed using the SYBR Green Supermix (Bio-Rad) on the CFX384™ real-time PCR detection system (Bio-Rad). The PCR-program included initialization (6 min at 95°C), 45 cycles of denaturation (15 s at 95°C), annealing (30 s at 60°C) and elongation (30 s at 72°C), final elongation for 1 min at 72°C and a DNA melting curve (55°C to 95°C through 0.2°C increments every 10s). All samples were tested in duplicate. Specificity of PCR products was evaluated by size in agarose gel electrophoresis followed by DNA sequence analysis. Serial dilutions of cDNA from spontaneously immortalized keratinocytes (own laboratory) and universal human reference RNA (Stratagene, Santa Clara, CA USA) were included to determine PCR-efficiencies.

**Table 6:** QPCR primers

**Gene**	**Accession number**	**Forward primer 5**^**′**^** > 3**^**′**^	**Reverse primer 5**^**′**^** > 3**^**′**^	**Amplicon size (bp)**
*Genes of interest*				
CCL27	NM_006664	GACTGTCACCTCCAGGCTTT	TCTCTTGGTGCTCAAACCAC	100
K17	NM_000422	CTGGAGCAGGAGATTGCCAC	GGGTGGTCACCGGTTCTTTC	88
MMP1	NM_002421	AGGTCTCTGAGGGTCAAGCA	CTGGTTGAAAAGCATGAGCA	111
MMP3	NM_002422	TGCTTTGTCCTTTGATGCTG	GGAAGAGATGGCCAAAATGA	135
MMP9	NM_004994	CCTGGAGACCTGAGAACCAA	ATTTCGACTCTCCACGCATC	103
MMP10	NM_002425	GTGGAGTTCCTGACGTTGGT	TCAATGGCAGAATCAACAGC	130
PI3	NM_002638	GACTGCCCAGGAATCAAGAA	CAGCAGGGACTTAGGACCAG	148
SERPINB4	NM_002974	CAAAGGGCAGTGGGAGAATA	CCTCCAGCAAGGCAAAATTA	131
TUBB3	NM_006086	CTCAGGGGCCTTTGGACATC	CCCTCCGTGTAGTGACCCTT	96
*Reference genes*				
ARPC2	NM_005731	TCCGGGACTACCTGCACTAC	GGTTCAGCACCTTGAGGAAG	96
BAT3	NM_004639	AAGAGACGCAAGACGATGCAG	TGTAGCTCTCCTGAACCTCTGG	151
RPS29	NM_001032	TATGTGCCGCCAGTGTTTCC	TGCCCCGGATAATCCTCTGA	92
ZNF410	NM_021188	GCTGTGGTAAGCAGTTTACTACAG	CTTGGGCTTCACAAAGGAAAGG	90

The stable expression of the reference genes was validated by analyzing their QPCR data using the geNORM method (M < 1.0, CV < 0.5) in the freely available qBase software [54,55]. QPCR data of the genes of interest were normalized based on the expression of the reference genes using the normalization factor from geNORM. Relative gene expression between the different sample groups was statistically analyzed in Excel, using a two-sided Student’s *T*-test assuming unequal variance. P-value ≤ 0.05 was considered significant.

## Abbreviations

AK: Actinic keratosis; BAF: B allele frequency; BCC: Basal cell carcinoma; BP: Biological processes; DEG: Differentially expressed gene; DEP: Differentially expressed probe; ECM: Extracellular matrix; FDR: False discovery rate; GWEA: Genome-wide expression analysis; HPV: Human papilloma virus; KEGG: Kyoto encyclopedia of genes and genomes; log_2_FC: log_2_ fold change; LOH: Loss of heterozygosity; NS: Normal skin; OTR: Organ transplant recipient; PCA: Principal component analysis; (P)GSEA: (parametric) geneset enrichment analysis; QPCR: Quantitative RT-PCR; RT-PCR: Reversed transcriptase polymerase chain reaction; SCC: Squamous cell carcinoma; TSG: Tumor suppressor gene; TF: Transcription factor; TFBS: Transcription factor binding-site; UVR: Ultraviolet radiation.

## Competing interests

The authors declare that they have no competing interests.

## Author’s contributions

LH carried out the experiments, performed statistical analyses and drafted the manuscript. SC carried out the QPCR experiments and drafted the manuscript; JNBB and HCW selected patients and collected the samples; LH, JNBB, FRG, RW, LM, CPT and HV designed the study; all authors saw and approved the final manuscript.

## Authors’ information

Cornelis P Tensen and Harry Vrieling shared senior authorship.

## Pre-publication history

The pre-publication history for this paper can be accessed here:

http://www.biomedcentral.com/1471-2407/13/58/prepub

## Supplementary Material

Additional file 1 **Results of the Toptable from the limma analysis.** Differentially expressed probes between SCC and NS, between AK and NS, between SCC and AK, and the progression list with FDR < 0.01 and log_2_FC > = 0.5, including the VST transformed RSN normalized data. Click here for file

Additional file 2 **Result DAVID analysis for the differentially expressed probes between the sample groups from the limma analysis with log**_**2**_**FC > 0.5 and FDR < 0.01.** (XLS 201 kb) Click here for file
